# Mr. Davis on Ergot of Rye

**Published:** 1829-10-01

**Authors:** 


XXII.

OBSERVATIONS ON THE USE OF THE ERGOT OF RYE. By Mr. DAVIS, Surgeon, Pershore.

In a short paper published in a recent Number of the MIDLAND REPORTER, we find some observations by Mr. Davis on the ergot, which we shall lay before our readers. He remarks that the ergot has strong claims to our attention, its powers being *sui generis*, and really wonderful. "Under favourable circumstances, it will bring on and end a labour in an hour, which might have gone on for days, thus saving the practitioner much harrassing anxiety, as well as rescuing the most amiable and interesting portion of our species from much mental distress and acute and unnecessary suffering, and in all human probability, in many instances, from an untimely grave." But Mr. Davis is far from recommending the ergot upon all occasions. He thinks its administration requires great caution, and that it is likely to do incalculable mischief when injudiciously given.

"1. It ought rarely to be given in first cases.

"2. It ought not to be given if the pelvis be not capacious.

"3. It ought not to be given unless the parts be well dilated.

"4. It ought chiefly to be given in cases where the labor is unusually tedious, in consequence of want of action in the uterus.

"5. In many cases of hæmorrhage during parturition, and in retention of the placenta.

"If the parts be properly dilated and lubricated, and if the uterus be acting feebly and inefficiently, one dose of ergot will, I am fully persuaded, in nine cases out of ten, end the labor satisfactorily within an hour. It would be highly improper to give it in cases of impaction; in any presentation, excepting a natural one, or where there is a want of proper proportion between the child's head and the pelvis of the mother; or where there exists any rigid contraction of the soft parts.

Mr. Davis has detailed six cases where the ergot was given with great advantage. In one case, it was a first labour, and the pains were dreadfully excruciating, and recurring with scarcely any intermission. Opium was repeatedly given without effect. The os uteri was dilated, but no advance was made. A single scruple of the ergot terminated the labour in an hour and a half.

